# Dual-Species Model Biofilm Consisting of *Listeria monocytogenes* and *Salmonella* Typhimurium: Development and Inactivation With Cold Atmospheric Plasma (CAP)

**DOI:** 10.3389/fmicb.2019.02524

**Published:** 2019-11-07

**Authors:** Marlies Govaert, Cindy Smet, James L. Walsh, Jan F. M. Van Impe

**Affiliations:** ^1^CPMF^2^, Flemish Cluster Predictive Microbiology in Foods, Ghent, Belgium; ^2^OPTEC, Optimization in Engineering Center-of-Excellence, KU Leuven, Ghent, Belgium; ^3^BioTeC, Chemical and Biochemical Process Technology and Control, Department of Chemical Engineering, KU Leuven, Ghent, Belgium; ^4^Department of Electrical Engineering and Electronics, University of Liverpool, Liverpool, United Kingdom

**Keywords:** cold atmospheric plasma (CAP), biofilms, inactivation, dual-species, single-species, *L. monocytogenes*, *S*. Typhimurium

## Abstract

Most environmental biofilms contain a variety of species. These species can establish cooperative and competitive interactions, possibly resulting in an increase or a decrease in antimicrobial resistance. Therefore, results obtained following inactivation of single-species biofilms by means of different technologies (e.g., Cold Atmospheric Plasma, CAP) should be validated for multi-species biofilms. First, a strongly adherent and mature *Listeria monocytogenes* and *S*. Typhimurium dual-species biofilm was developed by altering different incubation conditions, i.e., growth medium, incubation temperature, inoculum ratio of *L. monocytogenes* and *S*. Typhimurium cells, and incubation time. Adherence and maturity were quantified by means of optical density measurements and viable plate counts, respectively. Secondly, both the (1 day old) reference biofilm and a more mature 7 days old biofilm were treated for different CAP treatment times (0–30 min). Viable plate counts were again used to determine the (remaining) cell density. For both the biofilm development and inactivation, predictive models were applied to describe the growth/inactivation kinetics. Finally, the kinetics of the [1 and 7 day(s) old] dual-species biofilms were compared with those obtained for the corresponding single-species biofilms. Results implied that a strongly adherent and mature reference dual-species biofilm was obtained following 24 h of incubation at 25°C using 20-fold diluted TSB and an inoculum ratio of 1:1. Main observations regarding CAP inactivation were: (i) the dual-species biofilm age had no influence on the CAP efficacy, although a longer treatment time was required for the oldest biofilm, (ii) for the 1 day old biofilms, CAP treatment became less efficient for *S*. Typhimurium inactivation when this species was part of the dual-species biofilm, while *L. monocytogenes* inactivation was not influenced by the biofilm type, and (iii) for the 7 days old biofilms, CAP inactivation of both species became more efficient when they were part of the dual-species biofilms. It can be concluded that the efficacy of the CAP treatment is altered when cells become part of a dual-species biofilm, which is quite important with respect to a possible application of CAP for biofilm inactivation within the food industry.

## Introduction

A biofilm consists of microbial cells embedded in a matrix of self-produced extracellular polymeric substances (EPS) and is attached to a biotic or an abiotic surface (Bakke et al., 1984; Costerton et al., 1987; Garrett et al., 2008; Giaouris et al., 2014). The compounds present in the EPS matrix are dependent on the microbial species, however, the matrix generally contains exopolysaccharides, extracellular DNA, and proteins (Ciofu and Tolker-Nielsen, 2011). Biofilms are omnipresent in nature and in many industrial environments. Due to their high resistance toward currently applied cleaning methods for (abiotic) surfaces, they can cause both economic and health related problems such as contamination/spoilage of food products, impeded heat transfer in heat exchangers, and corrosion of surfaces (Kumar and Anand, 1998; Garrett et al., 2008; Barry and Kanematsu, 2015; Javaherdashti, 2015). Annually, this results in a worldwide cost of billions of euros (Yang et al., 2011). To reduce this high cost, extensive studies concerning (novel) highly effective methods for biofilm inactivation/removal are required.

To date, most biofilm-related studies have been focusing on single-species biofilms. These studies have extended our general knowledge concerning biofilm formation, structure, and resistance. However, in natural environments (outside of the lab), biofilms mostly contain a variety of species (Yang et al., 2011; Elias and Banin, 2012; Burmølle et al., 2014). Due to the cooperative interspecies interactions within this type of biofilms, they can become more resistant toward antimicrobial agents than single-species biofilms (e.g., due to an increased biomass and/or an altered composition of the EPS matrix). Consequently, within the food industry, this could result in an increased risk of (cross) contamination of food products following contact with an ineffectively cleaned/decontaminated surface (Yang et al., 2011; Elias and Banin, 2012). However, apart from cooperative behavior, there might as well be competition between the different species (Yang et al., 2011; Elias and Banin, 2012; Rendueles and Ghigo, 2012; Burmølle et al., 2014; Liu et al., 2016). In that case, if competition within the multi-species biofilm is the dominant mode of interaction, antagonistic effect can occur (e.g., due to the production of toxins by one of the species). As a consequence, the biofilm-associated cells might show an increased sensitivity toward inactivation methods such as the use of antimicrobial agents (Elias and Banin, 2012).

In previous studies, Cold Atmospheric Plasma (CAP) has been investigated as a novel method for inactivation of biofilms (e.g., Vleugels et al., 2005; Niemira et al., 2014, 2018; Ziuzina et al., 2015; Govaert et al., 2018a, 2019). Plasma is a partially or wholly ionized gas which consists of a large variety of (reactive) species, including ions (positive and negative), photons, free electrons, and activated neutral species (excited and radical) (Tendero et al., 2006; Kudra and Mujumdar, 2009; Banu et al., 2012; Fernández and Thompson, 2012; Lu et al., 2013; Bourke et al., 2017). The use of plasma has some advantages over the currently applied disinfection methods for abiotic surfaces such as the use of (hot) water and antimicrobial agents. CAP treatment is relatively fast, it can be created at a low temperature, no water or chemicals are required, most plasma components fade out immediately after treatment, and cells can be inactivated by multiple inactivation mechanisms (Banu et al., 2012; Fernández and Thompson, 2012). The plasma species can interact with the microbial cells, possibly resulting in (i) a damaged (outer) membrane, (ii) an altered structure and/or altered functional properties of the macromolecules, and (iii) a negative influence on the DNA (Misra et al., 2011; Fernández and Thompson, 2012; Puligundla and Mok, 2017). Due to these multiple inactivation mechanisms, CAP already proved to be a promising method for inactivation of strongly adherent and mature single-species biofilms developed by two important pathogenic species, i.e., *Listeria monocytogenes* (Gram positive) and *Salmonella* Typhimurium (Gram negative) (Govaert et al., 2019). Following 10 min of CAP treatment at optimal conditions, log-reductions up to 3.5 log_10_ (CFU/cm^2^) have been obtained for these specific biofilms (Govaert et al., 2019). However, as mentioned before, multi-species biofilms can promote antimicrobial resistance (Yang et al., 2011; Elias and Banin, 2012), possibly resulting in an overestimation of the efficacy of the CAP treatment. Therefore, one should be careful extrapolating previously obtained results for single-species biofilms to (more realistic) multi-species biofilms without validation.

In this study, both the development and the CAP inactivation of a mature and strongly adherent dual-species model biofilm were investigated. The biofilm consisted of two pathogenic species (i.e., *L. monocytogenes* and *S*. Typhimurium) and the effect of different (incubation) conditions on the adherence and maturity of the biofilm was determined. These conditions were: (i) growth medium, (ii) incubation temperature, (iii) inoculum ratio of *L. monocytogenes* and *S*. Typhimurium cells in the inoculum, and (iv) incubation time. Once the optimal conditions for the dual-species model biofilm development were established, the model biofilm was treated with CAP. Optimal (single-species) CAP treatment conditions were applied as determined in the research of Govaert et al. (2019) and biofilms were treated for different treatment times ranging between 0 and 30 min. Optical density (OD) measurements and viable plate counts were used to quantify the adherence and the (remaining) cell density of the biofilm, respectively. Predictive models were finally applied to determine the growth and inactivation kinetics.

## Materials and Methods

### Experimental Design

In the first part of this research, a dual-species model biofilm consisting of *L. monocytogene*s and *S*. Typhimurium was developed. In order to obtain a strongly adherent and mature model biofilm, different (incubation) conditions were altered, i.e., (i) growth medium, (ii) incubation temperature, (iii) inoculum ratio of *L. monocytogenes* and *S*. Typhimurium cells, and (iv) incubation time. The adherence of the biofilm at each of the different (incubation) conditions was quantified by means of crystal violet staining and subsequent optical density (OD) measurements. To determine the cell density/maturity of the biofilm, viable plate counts were used. General and selective media were applied to determine the total biofilm cell density and the contribution of each individual species (i.e., *L. monocytogenes* and *S*. Typhimurium) to this total cell density. In addition, the Baranyi and Roberts (1994) model was used to determine the growth kinetics. During this part of the study, generally three biological (independent) replicates were performed in order to comment on the influence of the different incubation conditions on the adherence and the maturity of the dual-species biofilm. Only for the adherence as function of time, five biological (independent) replicates were used.

In the second part of this study, the obtained dual-species model biofilm was treated with CAP at optimal CAP treatment conditions previously determined (Govaert et al., 2019). These conditions involved the use of a Dielectric Barrier Discharge (DBD) electrode, helium as working gas, and an input voltage of 21.88 V (which resulted in a high voltage signal of ~6.5 kV). Biofilms were treated for 0, 1, 2, 5, 7.5, 10, 15, 20, 25, and 30 min and the remaining cell density following CAP treatment was determined by means of viable plate counts. General and selective media, combined with the Geeraerd et al. (2000) model, were used to determine the CAP inactivation kinetics of the total population and those corresponding to each species separately. As for the biofilm development part, again three biological (independent) replicates were performed in order to comment on the CAP inactivation kinetics for dual-species biofilms.

### Microorganism and Pre-culture Conditions

In this research, *L. monocytogenes* LMG23775 (isolated from sausages) and *S*. Typhimurium LMG14933 (isolated from bovine liver), both acquired from the BCCM/LMG bacteria collection of Ghent University in Belgium, were used. Stock-cultures were stored at −80°C in Brain Heart Infusion broth (BHI, VWR International, Belgium) and Tryptic Soy Broth (TSB, Becton Dickinson, US), respectively, which were both supplemented with 20 (v/v) % glycerol (VWR International, Belgium). For every experiment, a purity plate was prepared by spreading a loopful of stock-culture onto a LB agar plate [Lennox LB agar (Becton Dickinson, US) supplemented with 5 g/L NaCl (Sigma-Aldrich, US)]. The purity plates for *L. monocytogenes* and *S*. Typhimurium were incubated for 24 h at 30 and 37°C, respectively.

Starting from the purity plates, pre-cultures were prepared by transferring one colony into an Erlenmeyer flask containing 20 mL of LB medium [Lennox LB broth (Becton Dickinson, US) supplemented with 5 g/L NaCl]. *L. monocytogenes* and *S*. Typhimurium pre-cultures were incubated for 24 h at 30 and 37°C, respectively. Following this incubation period, stationary phase cultures with a cell density of ~10^9^ CFU/mL were obtained.

### Biofilm Development Conditions

The stationary phase pre-cultures were used to develop a 100-fold diluted inoculum with a cell density of ~10^7^ CFU/mL. Dependent on the specific (incubation) conditions, different ratios of *L. monocytogenes* and *S*. Typhimurium pre-cultures were mixed and different dilution/growth media were applied to obtain this inoculum. The investigated pre-culture ratios (*L. monocytogenes* over *S*. Typhimurium) were 1:1, 1:2, 2:1, 1:3, and 3:1 and the selected dilution/growth media were BHI and 20-fold-diluted TSB (TSB/20), which proved to be the optimal media for single-species biofilm development by *L. monocytogenes* and *S*. Typhimurium, respectively (Govaert et al., 2018b).

To develop the biofilms, 1.2 mL of the inoculum was transferred to a small Petri dish made out of polystyrene (50 mm diameter, 9 mm height, Simport, Canada). After inoculation, Petri dishes were closed and gently shaken to make sure the inoculum covered the entire surface. Dependent on the applied (incubation) conditions, Petri dishes were incubated for 0–24 h at 25 or 30°C, which were the optimal temperatures for *S*. Typhimurium and *L. monocytogenes* single-species biofilm formation, respectively (Govaert et al., 2018b).

Once the optimal biofilm formation conditions were established during the first part of this study, this optimal set of (incubation) conditions was used during the second part of the research (i.e., for CAP inactivation of the dual-species biofilm) as reference model biofilm. However, apart from this reference biofilm, a more mature biofilm with a longer incubation period (i.e., 7 days) was CAP treated as well since previous research proved that *L. monocytogenes* and *S*. Typhimurium single-species biofilms became more resistant toward CAP treatment with an increasing biofilm age (Govaert et al., 2018a). For this 7 days old biofilm, the same growth medium, incubation temperature, and ratio of *L. monocytogenes* and *S*. Typhimurium cells were applied as established during the first part of this study.

### CAP Equipment and Biofilm Inactivation Procedure

As mentioned before, the dual-species model biofilms were CAP treated at the optimal plasma characteristics previously determined in the research of Govaert et al. (2019). This optimal combination involved (i) the use of a DBD electrode configuration, (ii) helium as working gas (purity 99.996 %; flow rate 4 L/min), (iii) an input voltage of 21.88 V (resulting in a high voltage signal of ~6.5 kV), and (iv) a frequency of 15 kHz. For a detailed overview of the characteristics of the applied DBD electrode, the authors refer to previously mentioned study.

For the CAP treatment, rinsed and dried biofilm samples (see section Quantification of Biofilm Cell Density/Maturity by Means of Viable Plate Counts) were placed in between the electrodes and the reactor chamber was flushed for 4 min to ensure a homogeneous gas mixture. After this, the high-voltage power source was energized to generate the plasma. Samples were treated up to 30 min and immediately after the treatment removed from the reactor chamber to determine the remaining cell density (see section Quantification of Biofilm Cell Density/Maturity by Means of Viable Plate Counts).

### Quantification of Biofilm Adherence by Means of Crystal Violet Staining

The method used for quantification of the biofilm adherence at each (incubation) condition has been discussed in detail in the research of Govaert et al. (2018b). Similar as for previously mentioned study, biofilms were considered to be strongly adherent if the OD of the sample was four times higher than the cut-off value (OD sample > 4 × OD_c_). Here, the cut-off value (OD_c_) is defined as the average OD of the controls (fresh growth medium without cells) plus three times the standard deviation of these negative controls (Stepanović et al., 2000).

### Quantification of Biofilm Cell Density/Maturity by Means of Viable Plate Counts

In the first part of this study, the biofilm cell density/maturity at the different (incubation) conditions was determined by means of viable plate counts. Following 0–24 h of incubation, biofilms were removed from the incubator and three times rinsed with phosphate buffered saline (PBS) solution to remove the remaining planktonic cells. After the rinsing procedure, biofilms were allowed to dry in the laminar flow cabinet. Next, 2 mL of sterile PBS solution was added to the rinsed and dried biofilms and a cell scraper (blade width 20 mm, Carl Roth GmbH+Co, Germany) was used to remove the biofilm from the surface. Serial decimal dilutions of the obtained cell suspension were prepared [0.85 (v/v) % NaCl] and plated on agar plates. For each of the serial dilutions, three drops of 20 μL were plated on general and selective media in order to examine the total biofilm cell density and the cell density of each individual species (i.e., *L. monocytogenes* and *S*. Typhimurium), respectively. Brain Heart Infusion agar (BHIA, BHI supplemented with 14 g/L biological agar, VWR Chemicals, Belgium) was used as general medium to determine the total cell density of the biofilm. PALCAM (VWR Chemicals, Belgium) and Xylose Lysine Deoxycholate Agar (XLDA, Merck & Co, USA) were used as selective media for *L. monocytogenes* and *S*. Typhimurium, respectively. On these selective media, only healthy cells are able to form colonies, so only these cells were taken into account while determining the cell density of the individual species. Therefore, a (slight) underestimation of the total number of *L. monocytogenes* and *S*. Typhimurium cells present in the biofilm could occur as a consequence of the induction of sub-lethal injury (Noriega et al., 2013; Govaert et al., 2019). Before counting the colonies, agar plates were incubated for (at least) 24 h at 30°C (BHIA and PALCAM) or 37°C (XLDA). The detection limit of the applied plate counting method was 3.9 ln(CFU/cm^2^) [or 1.7 log_10_(CFU/cm^2^)].

In the second part of this research, biofilms were treated with CAP following rinsing and drying (see section CAP Equipment and Biofilm Inactivation Procedure). Immediately after the CAP treatment, the cell density (on general and selective media) was determined according to the procedure described above. Nevertheless, in this case, drops of 100 μL were applied to be able to detect lower cell concentrations, i.e., the detection limit became equal to 1.0 log_10_(CFU/cm^2^) [or 2.3 ln(CFU/cm^2^)].

### Modeling and Parameter Estimation

In the first part of this research, the experimental data for the OD as function of time was fitted as described in Baka et al. (2015). For the cell densities as function of time, the growth model of Baranyi and Roberts (1994) was used (Equations 1–3).

(1) $$\begin{array}{l} \frac{dN\left(t\right)}{dt}=\left(\frac{Q}{1+Q}\right){\cdot \mu }_{max}\cdot \left(1-\frac{N\left(t\right)}{Nmax}\right)\cdot N\left(t\right) \end{array}$$

(2) $$\begin{array}{l} \frac{dQ\left(t\right)}{dt}=\mu_{max}\cdot Q\left(t\right) \end{array}$$

(3) $$\begin{array}{l} \lambda \cdot \mu_{max}=\ln\left(1+\frac{1}{Q\left(0\right)}\right) \end{array}$$

Since the experiments in this study were conducted at static conditions, the model was, however, simplified to Equations (4) and (5).

(4) $$\begin{array}{l} \ln\left(N\left(t\right)\right)=\ln\left(N\left(0\right)\right)+\mu_{max}\cdot A\left(t\right)-\ln\left(1+\frac{e^{\mu_{max}\cdot A\left(t\right)}-1}{e^{\left(N_{max}-N\left(0\right)\right)}}\right)\; \end{array}$$

(5) $$\begin{array}{l} A\left(t\right)=t+\frac{1}{\mu_{max}}\cdot \ln\left(\frac{e^{-\mu_{max}\cdot t}+Q\left(0\right)}{1+Q\left(0\right)}\right) \end{array}$$

Here, *N*(*t*) [CFU/cm^2^] is the cell density at time t [h], *N*(*0*) [CFU/cm^2^] is the initial cell density at time 0, *N*_*max*_ [CFU/cm^2^] is the maximum cell density, μ_*max*_ [1/h] is the maximum specific growth rate, *Q* [-] represents the physiological state of the cells, *Q*(0) is a measure of the initial physiological state of the cells, and λ [h] is the lag time of the cells.

The model of Geeraerd et al. (2000), describing a microbial inactivation curve consisting of a log-linear inactivation phase and a tail (Equation 6), was used to fit the experimental data obtained following CAP treatment of the biofilms.

(6) $$\begin{array}{l} N\left(t\right)=(N_{0}-N_{res})\cdot e^{-k_{max}\cdot t}+N_{res} \end{array}$$

Here, *N*(*t*) [CFU/cm^2^] is the cell density at time *t* [min], *N*_0_ [CFU/cm^2^] is the initial cell density, *N*_*res*_ [CFU/cm^2^] is a more resistant subpopulation, and *k*_*max*_ [1/min] is the maximum specific inactivation rate. Based on the difference between log_10_ *N*_0_ and log_10_ *N*_*res*_, the final log-reduction values (following 30 min of CAP treatment) were calculated.

The parameters of the Baranyi and Roberts (1994) and Geeraerd et al. (2000) model were estimated via the minimization of the sum of squared errors (SSE), using the lsqnonlin routine of the Optimization Toolbox of Matlab version R2016a (The Mathworks, Inc.). At the same time, standard errors of the parameter estimations were determined based on the Jacobian matrix. The Root Mean Squared Error (RMSE) served as an absolute measure of the goodness of the model to fit the actual obtained data.

### Statistical Analysis

In the first part of this study, analysis of variance (ANOVA) tests were performed to determine whether there were any significant differences amongst means of OD values or logarithmically transformed viable counts at the different (incubation) conditions. A confidence level of 95.0 % (α = 0.05) was applied and Fisher's Least Significant Difference (LSD) test was used to distinguish which means were significantly different from others. In addition, ANOVA tests were performed to determine whether there were significant differences between the estimated model parameters obtained for the cell density as function of time for each population type (i.e., total, *L. monocytogenes*, and *S*. Typhimurium).

In the second part of this research, ANOVA tests were again used to determine whether there were significant differences between the estimated model parameters (obtained at both biofilm ages) for the total population and those obtained for the individual populations (i.e., *L. monocytogenes* and *S*. Typhimurium). In addition, the inactivation kinetics observed for the dual-species biofilms were compared with those previously observed for the single-species biofilms (Govaert et al., 2019).

All statistical analyses were performed using the Statgraphics 18 software (Statistical Graphics, Washington, USA). Significant differences between sample values/estimated model parameters were indicated with different (uppercase) letters or numbers (e.g., “a,” “b,” “A,” “B,” “1,” “2,” …), with “a,” “A,” or “1” indicating the lowest value.

## Results

### Influence of the Growth Medium, the Incubation Temperature, and the Inoculum Ratio on the Adherence and the Cell Density of the Dual-Species Biofilm

**Figure 1:**
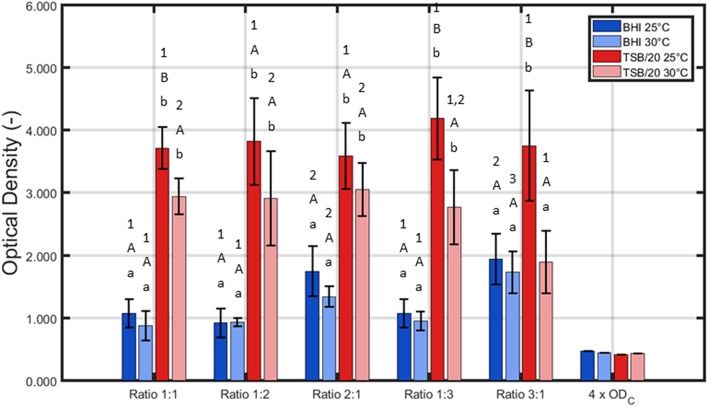
Influence of the incubation conditions (i.e., growth medium, incubation temperature, and the ratio of *L. monocytogenes* and *S*. Typhimurium in the inoculum) on the adherence (OD) of the dual-species biofilm (*n* = 3). Biofilms were incubated for 24 h prior to the quantification procedure and were considered to be strongly adherent as the average OD values were higher than their corresponding 4 × OD_C_-value. For the influence of the growth medium: for each temperature and each ratio, significant differences have been indicated by means of small letters, with “a” bearing the lowest value. For the influence of the incubation temperature: for each growth medium and each ratio, significant differences have been indicated by means of capital letters, with “A” bearing the lowest value. For the influence of the inoculum ratio: for each growth medium and each incubation temperature, significant differences have been indicated by means of numbers, with “1” bearing the lowest value.

**Figure 2:**
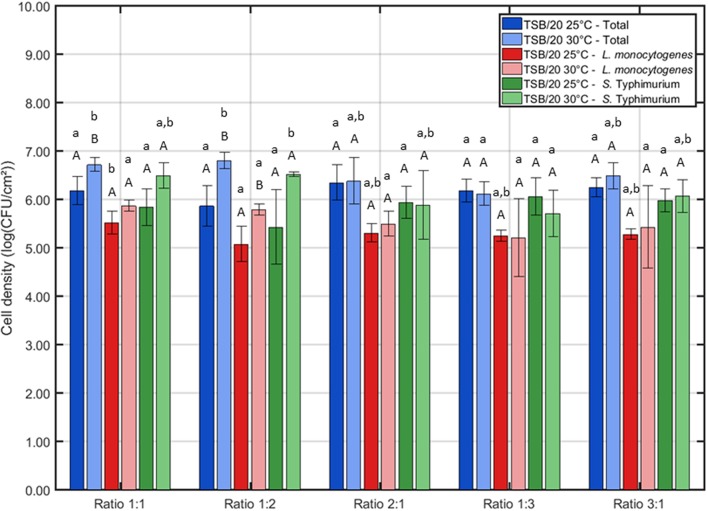
Influence of the incubation conditions (i.e., incubation temperature and the ratio of *L. monocytogenes* and *S*. Typhimurium in the inoculum) on the cell density of the dual-species biofilm (*n* = 3). Biofilms were incubated for 24 h prior to the quantification procedure. For the influence of the incubation temperature: for each ratio, significant differences have been indicated by means of capital letters, with “A” bearing the lowest value. For the influence of the inoculum ratio: for each incubation temperature, significant differences have been indicated by means of small letters, with “a” bearing the lowest value.

For the first series of experiments, the influence of the (incubation) conditions was tested for one certain incubation time, i.e., 24 h of incubation. This initial incubation period was selected based on previous research where 24 h of incubation proved to be optimal to obtain strongly adherent and mature single-species biofilms developed by *L. monocytogenes* and *S*. Typhimurium (Govaert et al., 2018b). The three other influencing factors (i.e., the growth medium, the incubation temperature, and the ratio of *L. monocytogenes* and *S*. Typhimurium in the inoculum) were altered to determine their effect on the adherence (Figure 1) and the cell density (Figure 2) of the dual-species biofilm. To determine if one incubation condition significantly influenced the adherence or cell density of the biofilm, different ANOVA tests were performed while keeping the other influencing factors constant.

Based on Figure 1, it can be concluded that for all tested conditions, strongly adherent biofilms were obtained since the average sample values were always higher than their corresponding 4 × OD_C_-value. Nevertheless, it should be stressed that all three altered incubation conditions significantly influenced the adherence of the dual-species biofilm. For the growth medium, the highest OD values were in general obtained using TSB/20. Only for one ratio (i.e., 3:1), no significant differences were observed between BHI and TSB/20 at 30°C. With respect to the influence of the incubation temperature, significant differences were only observed using TSB/20 as growth medium and at certain ratios (i.e., 1:1, 1:3, and 3:1). Here, the highest OD values were obtained using 25°C as incubation temperature. Finally, for the ratio of *L. monocytogenes* and *S*. Typhimurium, the influence on the adherence was again dependent on the other incubation conditions. Using BHI as growth medium, significantly higher OD values were observed while using ratios 2:1 and 3:1, and this for both temperatures. Using TSB/20 on the other hand, significant differences were only observed at 30°C. At this latter temperature, the ratio 3:1 resulted in significantly lower OD values compared to the ratios 1:1, 1:2, and 2:1.

Based on aforementioned results, it was decided to use only TSB/20 as growth medium for the investigation of the influence of the different (incubation) conditions on the cell density/maturity of the dual-species biofilm. Even though both TSB/20 and BHI resulted in the formation of strongly adherent biofilms, the OD values obtained using the former medium were at least two times higher than the corresponding values observed using the latter medium. As most biofilm studies relate the antimicrobial resistance of biofilm-associated cells to their incapsulation in the biofilm matrix (e.g., Costerton et al., 1987; Kumar and Anand, 1998), the higher biomass obtained using TSB/20 was deemed to result in a better protection of the biofilm-associated cells toward inactivation methods such as CAP. The effect of (i) the incubation temperature and (ii) the ratio of *L. monocytogenes* and *S*. Typhimurium in the inoculum on the biofilm cell density/maturity were further investigated, although 25°C tended to be more optimal at the ratios 1:1, 1:3, and 3:1.

Based on Figure 2, one can comment on the cell density of the biofilms following 24 h of incubation. It can be observed that the incubation temperature only had an influence on the cell density at two different ratios, i.e., 1:1 and 1:2. For these cases, the highest total cell density (and *L. monocytogenes* cell density for ratio 1:2) was obtained if the inoculated Petri dishes were incubated at 30°C. Regarding the influence of the inoculum ratio, only minor differences were observed. At 25°C, only the *L. monocytogenes* cell density was slightly influenced by this factor, i.e., the lowest cell density was obtained at ratio 1:2. At 30°C, the lowest cell densities were obtained at ratio 1:3 as here both the total cell density and the *S*. Typhimurium cell density were significantly lower than those obtained at all other ratios.

Combining the results obtained for the OD and the cell density of the dual-species biofilm, one incubation temperature and one ratio have been selected to further investigate the adherence and the cell density/maturity of the biofilm as function of the incubation time. For the temperature, 25°C was selected since this resulted for most ratios in the highest OD. As a more dense biofilm matrix might result in an increased resistance (Govaert et al., 2018a), this biofilm characteristic was deemed to be more important than the slightly higher cell densities observed at 30°C. For the ratio of the inoculum, 1:1 has been selected since at this ratio, the *L. monocytogenes* and *S*. Typhimurium cell densities appeared to be more or less equal following 24 h of incubation at 25°C. In conclusion, for the investigation of the adherence and the maturity of the dual-species biofilm as function of time, the following incubation conditions were selected: (i) TSB/20 as growth medium, (ii) 25°C as incubation temperature, and (iii) 1:1 as inoculum ratio.

### Influence of the Incubation Time on the Adherence and the Cell Density/Maturity of the Dual-Species Biofilm

**Figure 3:**
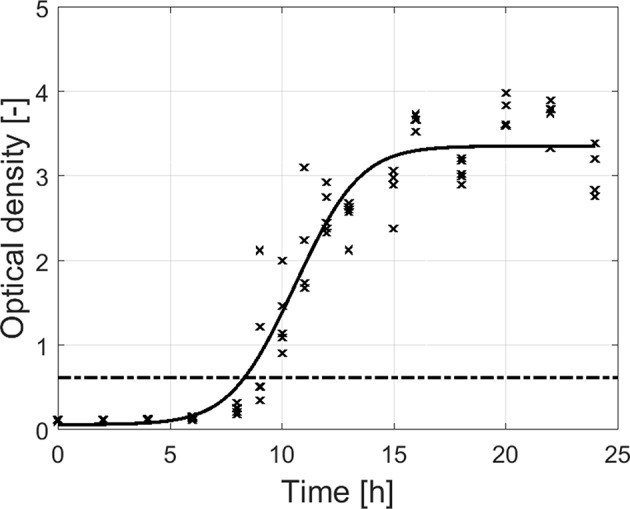
Adherence (OD) of the dual-species biofilm as function of the incubation time (*n* = 5). Biofilms were considered as strongly adherent if the OD of the sample exceeded the 4 × OD_C_-value (dash-dot line).

For the adherence of the biofilm as function of time (Figure 3), OD measurements were again used to quantify the total biofilm mass at each time point. An initial “lag phase” can be observed with a duration of ~8 h. During this phase, there was (almost) no increase in OD as function of time. However, upon further incubation, there is a steep increase in OD until ~14 h of incubation, indicating a strong increase in cell density and/or production of EPS matrix. Finally, this steep increase was followed by a plateau without further increase of the OD. Although this plateau was only observed following ~18 h of incubation, the dual-species biofilm became already strongly adherent starting from ~8.5 h of incubation (OD sample > 4 × OD_C_).

**Figure 4:**
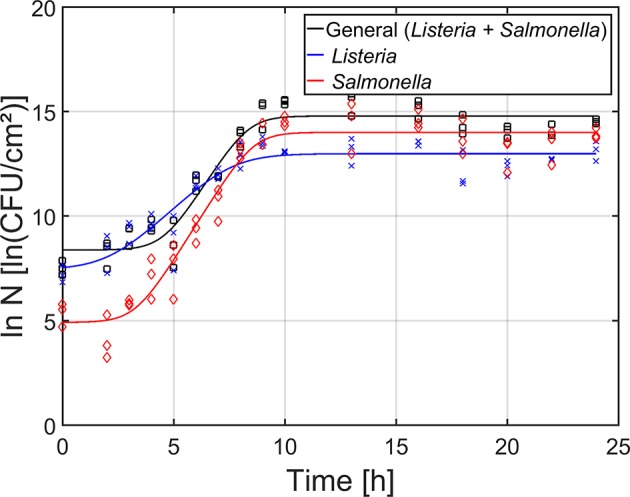
Maturity of the dual-species biofilm as function of time (*n* = 3). Both the experimental data (symbols) and the global fit (line) of the Baranyi and Roberts (1994) model are represented: total population on general medium (□, black line), *L. monocytogenes* population on Palcam medium (x, blue line), and *S*. Typhimurium population on XLD medium (◇, red line).

For the maturity of the biofilm as function of time (Figure 4), the total cell density as well as the cell densities of the individual species were determined by means of viable plate counts. The estimated model parameters of the Baranyi and Roberts (1994) model have been included in Table 1. It can be observed that starting from 0 h of incubation, some cells were already attached to the surface, i.e., the initial total cell density was 8.4 ln(CFU/cm^2^) and the initial cell densities for *L. monocytogenes* and *S*. Typhimurium were 7.5 and 4.9 ln(CFU/cm^2^), respectively. Despite the fast initial attachment of the cells, they did not immediately start to multiply, i.e., a (relatively short) lag-phase was observed for each of the population types (total: 4.6 h, *L. monocytogenes*: 2.3 h, and *S*. Typhimurium: 3.3 h). Upon further incubation, the total population as well as the population of the individual species started to increase. The specific growth rate for *S*. Typhimurium (1.7 h^−1^) was, however, higher than for *L. monocytogenes* (1.0 h^−1^). Finally, a stationary phase was observed for all population types, i.e., there was no further increase in cell density starting from ~10 h of incubation. In other words, the dual-species biofilm became mature following this incubation period. It should be mentioned, however, that the maximum cell density of *S*. Typhimurium [14.0 ln(CFU/cm^2^)] in the dual-species biofilm was higher than the maximum cell density of *L. monocytogenes* [13.0 ln(CFU/cm^2^)].

**Table 1:** Estimated parameters of the Baranyi and Roberts (1994) model for growth of the 1 day old dual-species biofilm.

**Model parameters**	**1 day old dual-species biofilm**
λ total (h)	4.56 ± 0.42^b^
*λ Listeria* (h)	2.25 ± 0.83^a^
*λ Salmonella* (h)	3.28 ± 0.45^a^
Ln *N_0_* total [ln(CFU/cm^2^)]	8.39 ± 0.42^c^
Ln *N_0_ Listeria* [ln(CFU/cm^2^)]	7.54 ± 0.42^b^
Ln *N_0_ Salmonella* [ln(CFU/cm^2^)]	4.93 ± 0.36^a^
*μ_*max*_* total (1/h)	1.70 ± 0.26^b^
*μ_*max*_ Listeria* (1/h)	1.04 ± 0.19^a^
*μ_*max*_ Salmonella* (1/h)	1.71 ± 0.18^b^
Ln *N_*max*_* total [ln(CFU/cm^2^)]	14.80 ± 0.16^c^
Ln *N_*max*_ Listeria* [ln(CFU/cm^2^)]	13.00 ± 0.17^a^
Ln *N_*max*_ Salmonella* [ln(CFU/cm^2^)]	14.02 ± 0.19^b^

*For each model parameter, a seperate ANOVA test has been performed to determine if these growth kinetics were influenced by the population type. Significant differences (p ≤ 0.05) have been indicated with a different lowercase letter, with ‘a' bearing the lowest value*.

Based on the combined results of the OD and the cell density as function of time, it can be concluded that strongly adherent and mature biofilms were already obtained following ~10 h of incubation. However, as the OD of the biofilm only became constant following ~18 h of incubation, a longer incubation period of 24 h was selected as optimal incubation time, similar to the research of Govaert et al. (2018b).

### CAP Inactivation of the Dual-Species Model Biofilm

#### Inactivation Kinetics of the 1 and 7 Day(s) Old Dual-Species Model Biofilm

**Figure 5:**
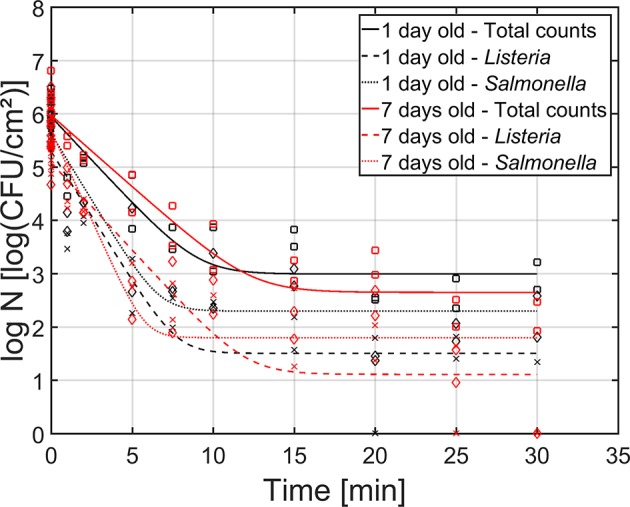
Cell density [log_10_ (CFU/cm^2^)] of the 1 (black) and 7 (red) day(s) old dual-species biofilm as function of the CAP treatment time (*n* = 3). Both the experimental data (symbols) and the global fit (line) of the Geeraerd et al. (2000) model are represented: total population on general medium (□, solid line), *L. monocytogenes* population on Palcam medium (x, dashed line), and *S*. Typhimurium population on XLD medium (♢, dotted line).

As mentioned before, both the reference biofilm (1 day old) and a more mature biofilm (7 days old) were CAP treated to examine (the influence of the biofilm age on) the inactivation kinetics for dual-species biofilms. In Figure 5, the cell density [log_10_(CFU/cm^2^)] of the 1 and 7 day(s) old dual-species biofilms as function of the CAP treatment time have been presented. The total population as well as the population of the individual species have been included. For each biofilm age and each population type (i.e., total, *L. monocytogenes*, and *S*. Typhimurium), the estimated model parameters of the Geeraerd et al. (2000) model have been included in Table 2.

**Table 2:** Estimated parameters of the Geeraerd et al. (2000) model for inactivation of the 1 and 7 day(s) old dual-species biofilms.

**Model parameters**	**1 day old dual-species biofilm**	**1 day old single-species biofilm**	**7 days old dual-species biofilm**	**7 days old single-species biofilms**
^2^ Log_10_ *N_0_* total_3_ ^1^ [log(CFU/cm^2^)]	^B^ 5.95 ± 0.15 ^a^	NA	^B^ 5.98 ± 0.13 ^a^	NA
^2^ Log_10_ *N_0_ Listeria*_3_ ^1^ [log(CFU/cm^2^)]	^A^ 5.30 ± ${0.21\;}_{1}^{\text{a}}$	7.15 ± 0.07 _2_	^A^ 5.09 ± ${0.19\;}_{1}^{\text{a}}$	6.22 ± 0.24 _2_
^2^ Log_10_ *N_0_ Salmonella*_3_ ^1^ [log(CFU/cm^2^)]	^A, B^ 5.63 ± ${0.20\;}_{1}^{\text{a}}$	6.14 ± 0.09 _2_	^B^ 5.69 ± ${0.24\;}_{1}^{\text{a}}$	5.40 ± 0.25 _1_
^2^ *k_*max*_* total_3_ ^1^ (1/min)	^A^ 0.75 ± 0.12 ^a^	NA	^A^ 0.62 ± 0.07 ^a^	NA
^2^ *k_*max*_ Listeria*_3_ ^1^ (1/min)	^A, B^ 1.12 ± ${0.24\;}_{1}^{\text{a}}$	1.74 ± 0.17 _2_	^A^ 0.76 ± ${0.12\;}_{2}^{\text{a}}$	0.28 ± 0.08 _1_
^2^ *k_*max*_ Salmonella*_3_ ^1^ (1/min)	^B^ 1.15 ± ${0.23\;}_{1}^{\text{a}}$	1.53 ± 0.20 _1_	^B^ 1.54 ± ${0.33\;}_{1}^{\text{a}}$	2.60 ± 0.69 _1_
^2^ Log_10_ *N_*res*_* total_3_ ^1^ [log(CFU/cm^2^)]	^C^ 3.00 ± 0.17 ^a^	NA	^C^ 2.65 ± 0.16 ^a^	NA
^2^ Log_10_ *N_*res*_ Listeria*_3_ ^1^ [log(CFU/cm^2^)]	^A^ 1.51 ± ${0.23\;}_{1}^{\text{a}}$	2.94 ± 0.13 _2_	^A^ 1.12 ± ${0.24\;}_{1}^{\text{a}}$	3.50 ± 0.56 _2_
^2^ Log_10_ *N_*res*_ Salmonella*_3_ ^1^ [log(CFU/cm^2^)]	^B^ 2.30 ± ${0.20\;}_{1}^{\text{b}}$	2.13 ± 0.16 _1_	^B^ 1.80 ± ${0.23\;}_{1}^{\text{a}}$	2.68 ± 0.17 _2_
^2^ Log-reduction total_3_ ^1^ [log(CFU/cm^2^)]	^A^ 2.95 ± 0.23 ^a^	NA	^A^ 3.32 ± 0.21 ^a^	NA
^2^ Log-reduction *Listeria*_3_ ^1^ [log(CFU/cm^2^)]	^B^ 3.79 ± ${0.31\;}_{1}^{\text{a}}$	4.22 ± 0.15 _1_	^B^ 3.97 ± ${0.31\;}_{2}^{\text{a}}$	2.72 ± 0.61 _1_
^2^ Log-reduction *Salmonella*_3_ ^1^ [log(CFU/cm^2^)]	^A, B^ 3.33 ± ${0.28\;}_{1}^{\text{a}}$	4.01 ± 0.18 _2_	^A, B^ 3.89 ± ${0.33\;}_{2}^{\text{a}}$	2.72 ± 0.30 _1_
RMSE total (-)	0.50	NA	0.44	NA
RMSE *Listeria* (-)	0.70	0.43	0.66	0.76
RMSE *Salmonella* (-)	0.65	0.55	0.78	0.63

1 *Influence biofilm age on dual-species biofilm: for each population type, model parameters bearing different superscripts (no small letters in common) are significantly different (P ≤ 0.05)*.

2 *Influence population type on dual-species biofilm: for each biofilm age, model parameters bearing different superscripts (no capital letters in common) are significantly different (P ≤ 0.05)*.

^3^*Influence biofilm type (dual-species vs. single-species): for each biofilm age, model parameters bearing different subscripts (no numbers in common) are significantly different (P ≤ 0.05)*.

Based on this figure, it can be observed that the shapes of the inactivation curves were similar for both biofilm ages, i.e., for all population types, a log-linear inactivation phase was followed by a tail. Based on Table 2, it can be concluded that the age of the dual-species biofilm generally did not influence the inactivation kinetics, i.e., only for the residual *S*. Typhimurium population, a significantly higher cell density was observed for the 1 day old biofilm than for the 7 days old biofilm. However, it should be stressed that, despite the lack of significant differences between the model parameters obtained for both biofilm ages, the required treatment time to obtain the residual (total) population appeared to be higher for the 7 days old biofilm than for the 1 day old biofilm (Figure 5). For the former biofilm age, ~17 min of CAP treatment was required, while for the latter biofilm age, ~12 min was sufficient to reach the residual cell density. Consequently, the efficacy of the CAP treatment was similar for both biofilm ages, but a longer treatment time was required (for the 7 days old biofilms) to obtain similar log-reduction values.

Regarding the effect of the population type (i.e., total, *L. monocytogenes*, and *S*. Typhimurium) on the inactivation kinetics, it can be observed that for the 1 day old dual-species biofilm, differences were only observed for the residual cell density, i.e., significantly lower values were obtained for *L. monocytogenes*. For the 7 days old dual-species biofilm, significant differences were observed for all model parameters, apart from the log-reduction values. For all these other parameters, significantly higher values were obtained for *S*. Typhimurium.

#### Comparison Between the Inactivation Kinetics and the Efficacy of CAP Treatment for Inactivation of Dual-Species and Single-Species Model Biofilms

**Figure 6:**
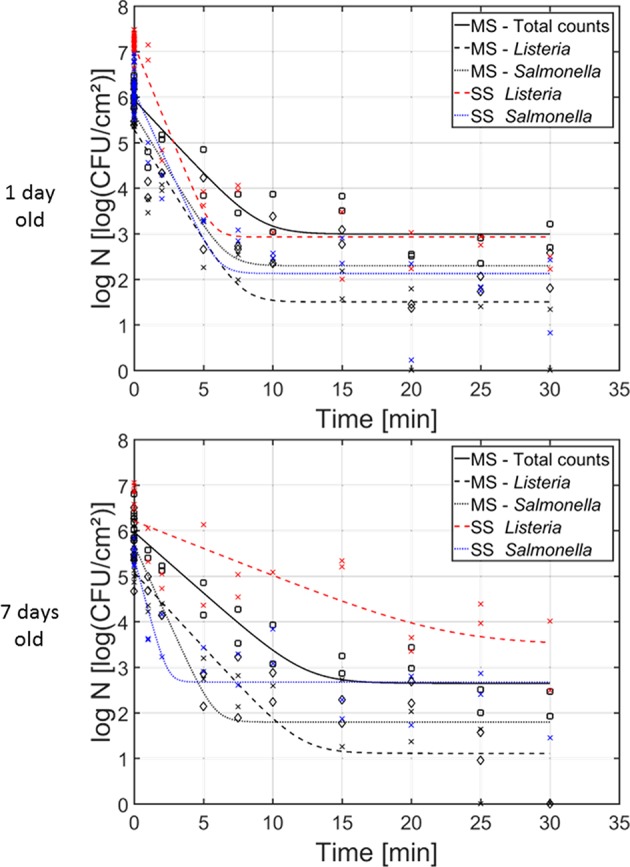
Cell density [log_10_ (CFU/cm^2^)] of the 1 and 7 day(s) old (i) dual-species biofilm (black), (ii) single-species *L. monocytogenes* biofilm (red), and (iii) single-species *S*. Typhimurium biofilm (blue) as function of the CAP treatment time (*n* = 3). Both the experimental data (symbols) and the global fit (line) of the Geeraerd et al. (2000) model are represented. For the dual-species biofilm: total population on general medium (□, solid line), *L. monocytogenes* population on Palcam medium (x, dashed line), and *S*. Typhimurium population on XLD medium (♢, dotted line). For the single-species biofilms: *L. monocytogenes* population on Palcam medium (x, dashed line) and *S*. Typhimurium population on XLD medium (x, dotted line).

In Figure 6, the cell density [log_10_(CFU/cm^2^)] of the (i) dual-species, (ii) *L. monocytogenes* single-species, and (iii) *S*. Typhimurium single-species biofilms as function of the CAP treatment time have been presented. Results have been included for both investigated biofilm ages, i.e., 1 and 7 day(s) old. For each model biofilm and each biofilm age, the estimated model parameters of the Geeraerd et al. (2000) model have been included in Table 2. The results obtained for the single-species biofilms (on selective media) have been presented in detail in the research of Govaert et al. (2018a).

For the 1 day old biofilms, it can be observed that the shape of the inactivation curves was again similar for all population and biofilm types, i.e., a log-linear inactivation phase was always followed by a residual population. The latter indicates that none of the biofilm types can be completely inactivated while applying the investigated (optimal) CAP treatment conditions. There were, however, some differences between the inactivation kinetics obtained for the individual populations of the dual-species biofilms and their corresponding inactivation kinetics obtained for the single-species biofilms. The initial cell density of the *L. monocytogenes* population in the dual-species biofilm is significantly lower than the corresponding value obtained for the *L. monocytogenes* single-species biofilm. In addition, also the inactivation rate and the residual cell density obtained for the *L. monocytogenes* population in the dual-species biofilm were significantly lower than the corresponding values obtained for the *L. monocytogenes* single-species biofilm. Nevertheless, when comparing the obtained log-reductions, the CAP treatment was equally effective for inactivation of *L. monocytogenes* in both biofilm types. For the *S*. Typhimurium population in the dual-species biofilm, significantly lower initial cell density values were obtained compared to the initial population of the single-species *S*. Typhimurium biofilm. Although there were no significant differences for the inactivation rate and the residual population, due to the difference in initial cell density (log_10_ *N*_0_), significantly higher log-reduction values were obtained for the single-species *S*. Typhimurium biofilm (in comparison to the *S*. Typhimurium population in the dual-species biofilm). As a consequence, it can be concluded that CAP treatment became less efficient for inactivation of the *S*. Typhimurium cells when they were part of the dual-species biofilm.

For the 7 days old biofilms, some significant differences were again observed. The initial *L. monocytogenes* cell density of the single-species biofilm was significantly higher than the corresponding cell density in the dual-species biofilm. The maximum inactivation rate, on the other hand, was significantly higher for *L. monocytogenes* in the dual-species biofilm than for the single-species biofilm. In addition, the residual cell densities for *L. monocytogenes* and *S*. Typhimurium in the dual-species biofilm were significantly lower than for the corresponding single-species biofilms. Consequently, the obtained log-reductions within the former biofilm type were also significantly higher than for the latter biofilm types, indicating an increased efficacy of the CAP treatment for both biofilm forming species when they are part of a mixed (7 days old) biofilm.

## Discussion

### Influence of the Growth Medium, the Incubation Temperature, and the Inoculum Ratio on the Adherence and the Cell Density of the Dual-Species Biofilm

In literature, biofilm formation has been reported to depend on different environmental conditions, including the growth medium, temperature, pH, incubation time, and oxygen tension (Gerstel and Römling, 2001; Stepanović et al., 2004; Harvey et al., 2007; Nilsson et al., 2011; Kadam et al., 2013; Nguyen et al., 2014; Tomičić et al., 2016; Govaert et al., 2018b). The influence of these environmental factors on the formation of biofilms is valid for single-species as well as multi-species biofilms. Marsh and Bowden (2000) reported for example that the ability of species to (i) attach to a surface, (ii) grow, and (iii) possibly dominate the community, is dependent on their habitat. Within the presented research, it was observed that the optimal dual-species biofilm formation conditions (following 24 h of incubation) included the use of (i) TSB/20 as growth medium, (ii) 25°C as incubation temperature, and (iii) 1:1 as ratio for *L. monocytogenes* and *S*. Typhimurium in the inoculum. Based on this, it can be concluded that *S*. Typhimurium predominantly determined the required environmental conditions since these optimal dual-species biofilm formation conditions were identical to previously observed optimal conditions required for single-species *S*. Typhimurium biofilm formation (Govaert et al., 2018b). Within this latter study, *L. monocytogenes* and *S*. Typhimurium biofilm formation required opposite nutritional requirements, i.e., a high vs. low nutrient content. Consequently, one could assume that within the dual-species biofilm, a certain form of metabolic cooperation was established, i.e., one species (*S*. Typhimurium) provided nutrients for the other (*L. monocytogenes*) (Bradshaw et al., 1997; Elias and Banin, 2012). In this case, byproducts formed by *S*. Typhimurium, as a consequence of its metabolic activity, could be used as a substrate for *L. monocytogenes*. Another possible explanation for *S*. Typhimurium not overgrowing *L. monocytogenes* could be that the initial attachment of *S*. Typhimurium cells to the surface facilitated the attachment and further growth of *L. monocytogenes*. Nevertheless, when the initial attachment of *L. monocytogenes* in the dual-species biofilm following 0 h of incubation (Figure 4) was compared with its initial attachment within the single-species biofilm (Govaert et al., 2018b), similar values were obtained for both biofilm types. Moreover, an additional test was performed to visualize the biofilm-associated cells by means of confocal laser scanning microscopy in combination with fluorescent Gram stains (results not shown). This indicated that on the bottom of the biofilm, mainly *L. monocytogenes* cells were present, while the top of the biofilm mainly consisted of *S*. Typhimurium cells. Consequently, the fact that *S*. Typhimurium does not completely overgrow *L. monocytogenes* is more likely the result of the first hypothesis, i.e., metabolic cooperation. As the observed (layered) microcolonies contained both cell types, the cells were most likely able to exchange nutrients (Elias and Banin, 2012). In addition, cells being in close proximity of each other is also required for inter- and intra-species communication since the maximum calling distance between cells (within a *Pseudomonas Putida* biofilm) has been reported to be 78 μm (Gantner et al., 2006). Co-aggregation of the cells could facilitate these interactions since Egland et al. (2004) reported that (within an oral dual-species biofilm) signaling mainly occurred within, rather than across, cell clusters. Nevertheless, more research would be required in order to confirm the claimed hypothesis.

Regarding the influence of the incubation temperature on the dual-species biofilm formation, *L. monocytogenes* already proved to be able to form strongly adherent single-species biofilms at 25°C (Govaert et al., 2018b). Therefore, the incubation temperature was deemed to be less important than the growth medium since both biofilm forming species should be capable of forming a biofilm at 25°C. This was indeed confirmed within the presented research, i.e., the influence of the incubation temperature on the OD and the cell density of the biofilm was not as pronounced as the influence of the growth medium (Figures 1, 2).

### Influence of the Incubation Time on the Adherence and the Cell Density/Maturity of the Dual-Species Biofilm

Based on the results obtained for the cell density as function of time (Figure 4 and Table 1) and on the additional confocal laser scanning microscopy experiment (results not shown), it can be concluded that *L. monocytogenes* can more easily attach to the polystyrene surface than *S*. Typhimurium. This can be the result of the specific interactions that are established between the polystyrene surface and the surface of the cells. As polystyrene is a hydrophobic surface, hydrophobic interactions are very important. The more hydrophobic the cells, the more likely they will adhere to the surface (Kochkodan et al., 2008; Giaouris et al., 2009). According to Sinde and Carballo (2000), *L. monocytogenes* and *Salmonella* spp. are both hydrophobic pathogens. However, the cell surface properties are not only dependent on the cell type (e.g., occurrence of cell appendages, the composition of the (outer) membrane, etc.), but also on the environmental conditions (e.g., temperature, pH, ionic strength, etc.) (Borecká-Melkusová and Bujdaková, 2008; Bujdakova et al., 2013). As the initial cell density (following 0 h of incubation) observed for *L. monocytogenes* was higher than for *S*. Typhimurium (Table 1), the cell surface properties were (within the presented research) more favorable for the former species. Another possible explanation for the lower initial adherence of the *S*. Typhimurium cells is related to the occurrence of competitive interactions. According to Rendueles and Ghigo (2012), certain species can produce biosurfactants, which are able to weaken bacteria-surface and bacteria-bacteria interactions. Consequently, the ability of other (competitive) species to form a biofilm is reduced. In addition, adhesion of certain species can be avoided/inhibited by another species due to surface blanketing. This latter phenomenon refers to the ability of certain species to occupy all available adhesion sites on a certain surface (Rendueles and Ghigo, 2012). Since *L. monocytogenes* mainly occurred in the lower layers of the biofilm, this could have resulted in an inhibited initial attachment of the *S*. Typhimurium cells.

Despite the (relatively fast) initial attachment of the cells, there was a lag phase observed for both the adherence (Figure 3) and the cell density (Figure 4) as function of time. This implies that there was (almost) no increase in cell density and (almost) no production of EPS matrix during the first hours of incubation. Since the “lag phase” for the adherence was slightly longer than for the cell density of the biofilm, it can be concluded that a minimal number of cells needs to be attached to the surface before they can be detected with the applied crystal violet staining method and/or before they start to produce (a significant) amount of EPS matrix. This latter phenomenon has been explained before by Jamal et al. (2015), i.e., biofilm-associated cells produce chemical signals upon replication and once a certain threshold is reached, the cells start to produce EPS.

Due to cell replication and by exceeding the chemical signal threshold, the initial lag phase was followed by a steep increase in cell density and OD (Figures 3, 4). The maximum specific growth rate (μ_*max*_) obtained for *S*. Typhimurium was higher than for *L*. monocytogenes (Table 1), which again indicates that the applied growth conditions were more favorable for the former biofilm species.

The steep increase observed for both the adherence and the cell density was followed by a plateau (Figures 3, 4). The incubation time to obtain this plateau was longer for the adherence than for the cell density, indicating that the cells continue to produce EPS matrix even after reaching their maximum cell density. Consequently, they can possibly become even more protected from stressing environmental conditions. The maximum cell density was, however, significantly higher for *S*. Typhimurium than for *L*. monocytogenes (Table 1), demonstrating again that the environmental conditions were slightly more favorable for the former biofilm forming species. Nevertheless, in comparison with the single-species maximum cell densities previously observed (Govaert et al., 2018b), it can be concluded that within the dual-species biofilm, lower *L. monocytogenes* and *S*. Typhimurium maximum cell densities (Table 1) were observed than within their corresponding single-species biofilm. This hints that, despite their ability to form a strongly adherent and mature dual-species biofilm, the conditions within this biofilm were in general less favorable for both species than within their corresponding single-species biofilm. Based on this, one can assume that within the dual-species biofilm, competitive effects are occurring as well. Cell growth can be inhibited as a consequence of waste accumulation, production of inhibitory agents, and/or nutrient limitations (Yang et al., 2011; Elias and Banin, 2012; Burmølle et al., 2014). In addition, dispersion of bacterial cells could have been induced as well by means certain compounds (e.g., matrix-degrading enzymes) produced by one of the species (Rendueles and Ghigo, 2012).

### CAP Inactivation of the Dual-Species Model Biofilm

Only a few prior studies focused on investigating the inactivation efficacy of CAP for multi-species biofilms. Three of these recent studies have been performed by Niemira et al. (2014), Modic et al. (2017), and Patanga et al. (2019). Within the first study, an air-based plasma jet was used to treat a multi-species biofilm containing three different *Salmonella* species. For the second research, an air-based plasma system was used to treat multi-species (and single-species) biofilms containing *Pseudomonas aeruginosa, Klebsiella pneumoniae, Enterococcus faecalis*, and *Staphylococcus aureus* cells. Finally, the third research investigated the inactivation of a dual-species biofilm containing *L. monocytogenes* and *Pseudomonas fluorescens* cells by means of an air-based plasma set-up. A direct comparison between the presented study and previously mentioned researches is, however, not possible due to the difference in applied (i) biofilm-forming species (and biofilm architecture) and (ii) plasma characteristics, of which the most important one is the operating gas (i.e., helium vs. air). In addition, previously mentioned studies did not all include a comparison between the CAP inactivation results/kinetics obtained for the multi-species biofilm and their corresponding single-species biofilms. Consequently, the findings observed within the presented research were mainly explained based on general knowledge regarding (multi-species) biofilms and their resistance toward traditionally used inactivation methods such as the use of antimicrobial agents.

#### Inactivation Kinetics of the 1 and 7 Day(s) Old Dual-Species Model Biofilm

Main observations regarding the inactivation kinetics of the 1 and 7 day(s) old dual-species model biofilms were: (i) for each population type and each biofilm age, all inactivation curves had a similar shape, (ii) the model parameters were influenced by the population type, but in general not by the biofilm age, and (iii) although the efficacy of the CAP treatment (based on log-reductions) did not decrease at an increased biofilm age, a longer treatment time was required to obtain similar log-reductions as for the reference (1 day old) biofilm.

All inactivation curves consisted of a log-linear phase followed by a tail (Figure 5). The tail phase implies the presence of a more resistant subpopulation, which cannot be inactivated with CAP (at the applied treatment conditions), even if the treatment time would be further increased. Since there was no shoulder phase observed, the destruction of the cells was faster than the internal repair mechanism of the cells, resulting in an immediate inactivation upon CAP treatment (Geeraerd et al., 2000).

Although the shapes of the inactivation curves were similar for all population types within the dual-species biofilms, some significant differences were observed between the model parameters obtained for *L. monocytogenes* and *S*. Typhimurium (for the same biofilm age) (Table 2). Nevertheless, despite these (significant) differences between the initial cell densities (log_10_ *N*_0_), inactivation rates (*k*_*max*_), and residual cell densities (log_10_ *N*_*res*_), the obtained log-reduction values for both biofilm forming species were similar. This is in contradiction to the research of Kostaki et al. (2012) were treatment of 6 days old *L. monocytogenes* and *S. enterica* dual-species biofilms with four different antimicrobial agents resulted in a higher inactivation of the latter species. Nevertheless, one should be careful comparing these results since (i) CAP inactivation involves a different inactivation mechanism, (ii) different biofilm development conditions were applied, and (iii) different strains were used. In previous studies, it was reported that the cells located in the bottom layers of a biofilm can become more resistant toward antimicrobial treatments due to a limited diffusion of the reactive components into the 3-dimensional biofilm matrix and/or a decreased metabolic activity of the cells located in the lower layers (Kumar and Anand, 1998). The similar log-reduction values obtained for both *L. monocytogenes* (mainly located in the bottom layers) and *S*. Typhimurium (mainly located in the top layers) indicate, however, that the diffusion of the plasma species into the dual-species biofilm matrix was not hindered.

The lack of significant differences between the model parameters obtained for the 1 and 7 day(s) old dual-species biofilms is (partially) in contradiction to previous research investigating the effect of the biofilm age on the resistance of single-species *L. monocytogenes* and *S*. Typhimurium biofilms toward CAP treatment (Govaert et al., 2018a). For the initial cell density (log_10_ *N*_0_) of these single-species biofilms, a decrease in biofilm-associated cells was observed as the biofilm age increased. This was deemed to be a consequence of biofilm-associated cells that died due to starvation and/or waste accumulation (Anwar et al., 1992). The absence of a decrease in initial cell density at an increased dual-species biofilm age implies that the cells within the dual-species biofilm are more protected against nutrient depletion and/or waste accumulation than their corresponding single-species biofilms. As mentioned before, this was possibly the result of a certain form of metabolic cooperation, where one species (i.e., *S*. Typhimurium) provides nutrients for the other species (i.e., *L. monocytogenes*) (Bradshaw et al., 1997; Elias and Banin, 2012). Nevertheless, other mechanisms could be involved as well, e.g., the composition of the EPS matrix can change as function of the biofilm age and/or a difference in gene expression can occur, both resulting in an altered biofilm resistance. The lack of an effect of the biofilm age on the CAP inactivation efficacy (based on log-reductions) is in compliance to the research of Niemira et al. (2014), while it is again partially in contradiction to the research of Govaert et al. (2018a). Within the former study, the CAP efficacy for inactivation of a multi-species biofilm containing three different *Salmonella* species proved not to be influenced by the biofilm age. Within the latter study, on the other hand, the efficacy of the treatment following 30 min of CAP treatment decreased at an increased single-species *S*. Typhimurium biofilm age, while it remained the same for the *L. monocytogenes* biofilm. Nevertheless, for the latter biofilm forming species, an increased CAP treatment time was required to obtain similar log-reductions for both tested biofilm ages [i.e., 1 and 7 day(s)]. This is in compliance to currently presented research as an increased treatment time was required for the 7 days old dual-species biofilm to obtain a similar log-reduction as for the 1 day old reference dual-species biofilm. This increased treatment time is very important to take into account as this could result in an increased risk of cross-contamination following a long period of equipment downtime. More research would again be required [e.g., by studying the penetration of plasma species within the 1 and 7 day(s) old biofilms] to determine the cause of this required increase in treatment time.

#### Comparison Between the Inactivation Kinetics and the Efficacy of CAP Treatment for Inactivation of Dual-Species and Single-Species Model Biofilms

When the inactivation kinetics obtained for the 1 day old dual-species biofilm were compared with those obtained for the corresponding single-species biofilms, most important findings were the fact that (i) significantly lower initial (*L. monocytogenes* and *S*. Typhimurium) cell densities were obtained within the dual-species biofilms, (ii) the residual *L. monocytogenes* cell density was significantly lower within the dual-species biofilm, and (iii) the CAP efficacy (based on log-reductions) for inactivation of *L. monocytogenes* was similar for both biofilm types, while the efficacy for *S*. Typhimurium inactivation was significantly higher within the dual-species biofilm. For the 7 days old biofilms, on the other hand, it was concluded that (i) the initial cell density within the dual-species biofilms was only significantly lower for *L. monocytogenes*, (ii) the residual cell densities were for both species significantly lower when they were part of the dual-species biofilm, and (iii) the obtained log-reductions were for both biofilm forming species significantly higher within the dual-species biofilm than for their corresponding single-species biofilms.

Although *L. monocytogenes* and *S*. Typhimurium were clearly able to form strongly adherent and mature [1 and 7 days(s) old] dual-species model biofilms, the significantly lower initial cell densities observed for the dual-species biofilms (in comparison to their corresponding single-species biofilms) again indicate that the environmental conditions within the dual-species biofilm were for both species not equally optimal as when they formed single-species biofilms. As mentioned before, *L. monocytogenes* required a high nutrient concentration at 30°C in order to develop a strongly adherent and mature single-species biofilm (Govaert et al., 2018b). Consequently, although metabolic cooperation might have occurred, the limited nutrient concentration of the dual-species growth medium possibly limited the *L. monocytogenes* growth. For *S*. Typhimurium, the limited growth could have been caused as well by nutrient depletion. However, as optimal single-species *S*. Typhimurium biofilm formation was also obtained while using a similar nutrient poor growth medium, waste accumulation/product inhibition was more likely the cause of the limited *S*. Typhimurium growth. In addition, *S*. Typhimurium cell dispersion could have been induced by *L. monocytogenes* in order to limit the (competitive) nutrient uptake of the *S*. Typhimurium cells (Rendueles and Ghigo, 2012).

Regarding the residual cell densities obtained following CAP treatment of the 1 and 7 days old model biofilms, in general lower values were obtained for the dual-species model biofilms. Only for the 1 day old biofilms, the biofilm type had no influence on the residual *S*. Typhimurium cell density. Consequently, this general observation indicates that the *L. monocytogenes* and *S*. Typhimurium cells became less resistant toward CAP treatment when they were part of the dual-species biofilms. A possible explanation for this phenomenon can be found in the study of Elias and Banin (2012). Although most multi-species biofilms generally exhibit an increased resistance toward antibiotics and antimicrobial agents, competition within a mixed biofilm can result in an increased sensitivity of the cells, i.e., if (one of) the species produced bacteriocins, addition of an external antimicrobial agent can result in a highly effective inactivation.

In contrast, the (significantly) lower log-reductions obtained for *L. monocytogenes* and *S*. Typhimurium within the 1 day old dual-species biofilm (in comparison with the corresponding single-species biofilms) indicate that CAP treatment becomes less effective when the cells are part of a dual-species biofilm. This observation can be related, however, to the lower initial cell densities [log_10_(*N*_0_)] obtained for *L. monocytogenes* and *S*. Typhimurium present within the dual-species biofilm. For the 7 days old dual-species biofilms, on the other hand, the lower residual cell densities observed following CAP treatment of the dual-species biofilm resulted as well in higher log-reductions obtained. Consequently, CAP treatment becomes more effective when the *L. monocytogenes* and *S*. Typhimurium cells are present within the (7 days old) dual-species biofilm. As mentioned before, this can be explained based on the possible production of bacteriocins, resulting in an overall higher efficacy of the CAP treatment.

Although some hypotheses regarding the specific effect of CAP on the 1 and 7 day(s) (dual-species) model biofilms have been formulated within this section, further research would be required to confirm these theories. Future studies could, for example, focus on investigating (i) the composition of the generated plasma (type and quantity of reactive species), (ii) the specific 3-dimensional structure of each model biofilm, (iii) the ability of the different plasma species to penetrate into the different model biofilms, and (iv) phenotypic changes occurring within the *L. monocytogenes* and *S*. Typhimurium cells due to their growth within the different model biofilms. In addition, as the CAP treatment conditions applied within this study were optimal for single-species biofilm inactivation (Govaert et al., 2019), further optimization of the CAP treatment conditions for more complex model biofilms could be examined. This is of high importance since the efficacy of the CAP treatment was, similar as for biofilm treatment with antimicrobial agents (Costerton et al., 1987; Kumar and Anand, 1998; Jefferson, 2004; Giaouris et al., 2014), not sufficiently high to obtain complete inactivation.

## Conclusions

A strongly adherent and mature reference dual-species biofilm was obtained following 24 h of incubation at 25°C using 20-fold diluted TSB and an inoculum ratio of 1:1. Initially, attachment of the *L. monocytogenes* cells was higher than for the corresponding *S*. Typhimurium cells, which was the result of the specific environmental conditions promoting (hydrophobic) interactions between the *L. monocytogenes* cells and the polystyrene surface. Nevertheless, the *S*. Typhimurium cell density of the mature dual-species biofilm proved to be higher than for *L. monocytogenes*, which was caused by competitive interactions between the species. Main conclusions regarding CAP inactivation were: (i) the dual-species biofilm age had no influence on the CAP efficacy, although a longer treatment time was required for the oldest biofilm, (ii) for the 1 day old biofilms, CAP treatment became less efficient for *S*. Typhimurium inactivation when this species was part of the dual-species biofilm, while *L. monocytogenes* inactivation was not influenced by the biofilm type, and (iii) for the 7 days old biofilms, CAP inactivation of both species became more efficient when they were part of the dual-species biofilm. These observations were caused by competitive and cooperative interactions between the species, differences in 3-dimensional biofilm structure, differences in EPS matrix composition, and/or phenotypical changes. As the efficacy of the CAP treatment proved to be influenced by the biofilm-type (i.e., single-species vs. dual-species) and the biofilm age, this should be taken into account with respect to a possible application of CAP within the food industry.

## Data Availability Statement

The datasets generated for this study are available on request to the corresponding author.

## Author Contributions

MG, CS, and JV: conceptualization and supervision. MG and CS: methodology, validation, and formal analysis. MG: software, investigation, data curation, writing-original draft preparation, visualization, and project administration. JW and JV: resources. MG, CS, JW, and JV: writing-review and editing. JV: funding acquisition.

### Conflict of Interest

The authors declare that the research was conducted in the absence of any commercial or financial relationships that could be construed as a potential conflict of interest.

## References

[B1] Anwar H. Strap J. L. Costerton J. W. (1992). Establishment of aging biofilms: possible mechanism of bacterial resistance to antimicrobial therapy. Antimicrob Agents Chemother. 36, 1347–1351. 10.1128/AAC.36.7.1347 1510427 PMC191585

[B2] Baka M. Noriega E. Stamati I. Logist F. Van Impe J. F. M. (2015). Critical assessment of the time-to-detection method for accurate estimation of microbial growth parameters. J. Food SAF 35, 179–192. 10.1111/jfs.12170

[B3] Bakke R. Trulear M. G. Robinson J. A. Characklis W. G. (1984). Activity of *Pseudomonas aeruginosa* in biofilms: steady state. Biotechnol. Bioeng. 26, 1418–1424. 10.1002/bit.260261204 18551671

[B4] Banu M. S. Sasikala P. Dhanapal A. Kavitha V. Yazhini G. Rajamani L. (2012). Cold plasma as a novel food processing technology. Annu. Rev. 4, 803–818.

[B5] Baranyi J. Roberts T. A. (1994). A dynamic approach to predicting bacterial growth in food. Int. J. Food Microbiol. 23, 277–294. 10.1016/0168-1605(94)90157-0 7873331

[B6] Barry D. M. Kanematsu H. (2015). Cooling water, in Biofilm and Materials Science, eds Kanematsu H. Barry D. M. (Cham: Springer International Publishing), 79–84. 10.1007/978-3-319-14565-5_10

[B7] Borecká-Melkusová S. Bujdaková H. (2008). Variation of cell surface hydrophobicity and biofilm formation among genotypes of *Candida albicans* and *Candida dubliniensis* under antifungal treatment. Can. J. Microbiol. 54, 718–724. 10.1139/W08-060 18772934

[B8] Bourke P. Ziuzina D. Han L. Cullen P. J. Gilmore B. F. (2017). Microbiological interactions with cold plasma. J. Appl. Microbiol. 123, 308–324. 10.1111/jam.13429 28245092

[B9] Bradshaw D. J. Marsh P. D. Watson G. K. Allison C. (1997). Oral anaerobes cannot survive oxygen stress without interacting with facultative/aerobic species as a microbial community. Lett. Appl. Microbiol. 25, 385–387. 10.1111/j.1472-765X.1997.tb00001.x

[B10] Bujdakova H. Didiasova M. Drahovska H. Cernakova L. (2013). Role of cell surface hydrophobicity in *Candida albicans* biofilm. Central Eur. J. Biol. 8, 259–262. 10.2478/s11535-013-0136-y

[B11] Burmølle M. Ren D. Bjarnsholt T. Sørensen S. J. (2014). Interactions in multispecies biofilms: do they actually matter? Trends Microbiol. 22, 84–91. 10.1016/j.tim.2013.12.004 24440178

[B12] Ciofu O. Tolker-Nielsen T. (2011). Antibiotic tolerance and resistance in biofilms, in Biofilms Infections, eds Bjarnsholt T. Jensen P. Ø. Moser C. Høiby N. (New York, NY: Springer), 215–229. 10.1007/978-1-4419-6084-9_13

[B13] Costerton J. W. Cheng K. J. Geesey G. G. Ladd T. I. Nickel J. C. Dasgupta M.. (1987). Bacterial biofilms in nature and disease. Annu. Rev. Microbiol. 41, 435–464. 10.1146/annurev.mi.41.100187.002251 3318676

[B14] Egland P. G. Palmer R. J. Kolenbrander P. E. (2004). Interspecies communication in *Streptococcus gordonii*-*Veillonella atypica* biofilms: signaling in flow conditions requires juxtaposition. Proc. Natl. Acad. Sci. U.S.A. 101, 16917–16922. 10.1073/pnas.0407457101 15546975 PMC534724

[B15] Elias S. Banin E. (2012). Multi-species biofilms: living with friendly neighbors. FEMS. Microbiol. Rev. 36, 990–1004. 10.1111/j.1574-6976.2012.00325.x 22229800

[B16] Fernández A. Thompson A. (2012). The inactivation of *Salmonella* by cold atmospheric plasma treatment. Food. Res. Int. 45, 678–684. 10.1016/j.foodres.2011.04.009

[B17] Gantner S. Schmid M. Dürr C. Schuhegger R. Steidle A. Hutzler P.. (2006). *In situ* quantitation of the spatial scale of calling distances and population density-independent N-acylhomoserine lactone-mediated communication by rhizobacteria colonized on plant roots. FEMS Microbiol. Ecol. 56, 188–194. 10.1111/j.1574-6941.2005.00037.x 16629749

[B18] Garrett T. R. Bhakoo M. Zhang Z. (2008). Bacterial adhesion and biofilms on surfaces. Prog. Nat. Sci. 18, 1049–1056. 10.1016/j.pnsc.2008.04.001

[B19] Geeraerd A. H. Herremans C. H. Van Impe J. F. (2000). Structural model requirements to describe microbial inactivation during a mild heat treatment. Int. J. Food. Microbiol. 59, 185–209. 10.1016/S0168-160500362-7 11020040

[B20] Gerstel U. Römling U. (2001). Oxygen tension and nutrient starvation are major signals that regulate *agfD* promoter activity and expression of the multicellular morphotype in *Salmonella* Typhimurium. Environ. Microbiol. 3, 638–648. 10.1046/j.1462-2920.2001.00235.x 11722544

[B21] Giaouris E. Chapot-Chartier M. Briandet R. (2009). Surface physicochemical analysis of natural *Lactococcus lactis* strains reveals the existence of hydrophobic and low charged strains with altered adhesive properties. Int. J. Food. Microbiol. 131, 2–9. 10.1016/j.ijfoodmicro.2008.09.006 18954916

[B22] Giaouris E. Heir E. Hébraud M. Chorianopoulos N. Langsrud S. Møretrø T.. (2014). Attachment and biofilm formation by foodborne bacteria in meat processing environments: causes, implications, role of bacterial interactions and control by alternative novel methods. Meat. Sci. 97, 298–309. 10.1016/j.meatsci.2013.05.023 23747091

[B23] Govaert M. Smet C. Baka M. Ecimovic B. Walsh J. L. Van Impe J. (2018a). Resistance of *L. monocytogenes* and *S*. Typhimurium towards cold atmospheric plasma as function of biofilm age. Appl. Sci. 8:2702 10.3390/app8122702

[B24] Govaert M. Smet C. Baka M. Janssens T. Van Impe J. (2018b). Influence of incubation conditions on the formation of model biofilms by *Listeria monocytogenes* and *Salmonella* Typhimurium on abiotic surfaces. J. Appl. Microbiol. 125, 1890–1990. 10.1111/jam.14071 30117654

[B25] Govaert M. Smet C. Vergauwen L. Ećimović B. Walsh J. L. Baka M. (2019). Influence of plasma characteristics on the efficacy of Cold Atmospheric Plasma (CAP) for inactivation of *Listeria monocytogenes* and *Salmonella* Typhimurium biofilms. Innovat. Food Sci. Emerg. Technol. 52, 376–386. 10.1016/j.ifset.2019.01.013

[B26] Harvey J. Keenan K. P. Gilmour A. (2007). Assessing biofilm formation by *Listeria monocytogenes* strains. Food. Microbiol. 24, 380–392. 10.1016/j.fm.2006.06.006 17189764

[B27] Jamal M. Tasneem U. Hussain T. Andleeb S. (2015). Bacterial biofilm: its composition, formation and role in human infections. Res. Rev. J. Microbiol. Biotechnol. 4, 1–14.

[B28] Javaherdashti R. (2015). Corrosion and biofilm, in Biofilm and Materials Science, eds Kanematsu H. Barry D. M. (Cham: Springer International Publishing), 69–78. 10.1007/978-3-319-14565-5_9

[B29] Jefferson K. K. (2004). What drives bacteria to produce a biofilm? FEMS Microbiol. Lett. 236, 163–173. 10.1016/j.femsle.2004.06.005 15251193

[B30] Kadam S. R. den Besten H. M. van der Veen S. Zwietering M. H. Moezelaar R. Abee T. (2013). Diversity assessment of *Listeria monocytogenes* biofilm formation: impact of growth condition, serotype and strain origin. Int. J. Food. Microb. 165, 259–264. 10.1016/j.ijfoodmicro.2013.05.025 23800738

[B31] Kochkodan V. Tsarenko S. Potapchenko N. Kosinova V. Goncharuk V. (2008). Adhesion of microorganisms to polymer membranes: a photo bactericidal effect of surface treatment with TiO_2_. Desalination 220, 380–385. 10.1016/j.desal.2007.01.042

[B32] Kostaki M. Chorianopoulos N. Braxou E. Nychas G. J. Giaouris E. (2012). Differential biofilm formation and chemical disinfection resistance of sessile cells of *Listeria monocytogenes* strains under monospecies and dual-species (with *Salmonella enterica*) conditions. Appl. Environ. Microbiol. 78, 2586–2595. 10.1128/AEM.07099-11 22307304 PMC3318796

[B33] Kudra T. Mujumdar A. S. (2009). Advanced Drying Technologies. Boca Raton, FL: CRC Press 10.1201/9781420073898

[B34] Kumar G. C. Anand S. K. (1998). Significance of microbial biofilms in food industry: a review. Int. J. Food. Microbiol. 42, 9–27. 10.1016/S0168-160500060-9 9706794

[B35] Liu W. Røder H. L. Madsen J. S. Bjarnsholt T. Sørensen S. J. Burmølle M. (2016). Interspecific bacterial interactions are reflected in multispecies biofilm spatial organization. Front. Microbiol. 7:1366. 10.3389/fmicb.2016.01366 27630624 PMC5005372

[B36] Lu H. Patil S. Keener K. M. Cullen P. J. Bourke P. (2013). Bacterial inactivation by high voltage atmospheric cold plasma: influence of process parameters and effects on cell leakage and dna. J. Appl. Microbiol. 116, 784–794. 10.1111/jam.12426 24372804

[B37] Marsh P. D. Bowden G. H. (2000). Microbial community interactions in biofilms, in Community Structure and Co-operation in Biofilms, eds Allison D. G. Gilbert P. Lappin-Scott H. Wilson M. (Cambridge: Cambridge University Press), 167–198. 10.1017/CBO9780511754814.010

[B38] Misra N. N. Tiwari B. K. Raghavarao K. S. Cullen P. J. (2011). Nonthermal Plasma Inactivation of Food-Borne Pathogens. Food. Eng. Rev. 3, 159–170. 10.1007/s12393-011-9041-9

[B39] Modic M. McLeod N. P. Sutton J. M. Walsh J. L. (2017). Cold atmospheric pressure plasma elimination of clinically important single- and mixed-species biofilms. Int. J. Antimicrob. Agents 49, 375–378. 10.1016/j.ijantimicag.2016.11.022 28161488

[B40] Nguyen H. D. N. Yang Y. S. Yuk H. G. (2014). Biofilm formation of *Salmonella* Typhimurium on stainless steel and acrylic surfaces as affected by temperature and pH level. Food. Sci. Technol. 55, 383–388. 10.1016/j.lwt.2013.09.022

[B41] Niemira B. A. Boyd G. Sites J. (2014). Cold plasma rapid decontamination of food contact surfaces contaminated with *Salmonella* biofilms. J. Food. Sci. 79, M917–M922. 10.1111/1750-3841.12379 24749764

[B42] Niemira B. A. Boyd G. Sites J. (2018). Cold plasma inactivation of *Escherichia coli* O17:H7 biofilms. Front. Sustain. Food. Syst. 2:47 10.3389/fsufs.2018.00047

[B43] Nilsson R. E. Ross T. Bowman J. P. (2011). Variability in biofilm production by *Listeria monocytogenes* correlated to strain origin and growth conditions. Int. J. Food. Microbiol. 150, 14–24. 10.1016/j.ijfoodmicro.2011.07.012 21824672

[B44] Noriega E. Velliou E. Van Derlinden E. Mertens L. Van Impe J. F. (2013). Effect of cell immobilization on heat-induced sublethal injury of *Escherichia coli, Salmonella* Typhimurium and *Listeria innocua*. Food Microbiol. 36, 355–364. 10.1016/j.fm.2013.06.015 24010617

[B45] Patanga A. Boehm D. Ziuzina D. Cullen P. J. Gilmore B. Bourke P. (2019). High voltage atmospheric cold air plasma control of bacterial biofilms on fresh produce. Int. J. Microbiol. 293, 137–145. 10.1016/j.ijfoodmicro.2019.01.005 30711711

[B46] Puligundla P. Mok C. (2017). Potential applications of nonthermal plasmas against biofilm-associated micro-organisms *in vitro*. J. Appl. Microbiol. 122, 1134–1148. 10.1111/jam.13404 28106311

[B47] Rendueles O. Ghigo J.-M. (2012). Multi-species biofilms: how to avoid unfriendly neighbors. FEMS Microbiol. Rev. 36, 972–989. 10.1111/j.1574-6976.2012.00328.x 22273363

[B48] Sinde E. Carballo J. (2000). Attachment of *Salmonell*a spp. and Listeria monocytogenes to stainless steel, rubber and polytetrafluorethylene: the influence of free energy and the effect of commercial sanitizers. Food Microbiol. 17, 439–447. 10.1006/fmic.2000.0339

[B49] Stepanović S. Cirković I. Ranin L. Švabić-Vlahović M. (2004). Biofilm formation by *Salmonella* spp. *and Listeria monocytogenes* on plastic surface. Lett. Appl. Microbiol. 38, 428–432. 10.1111/j.1472-765X.2004.01513.x 15059216

[B50] Stepanović S. Vuković D. Dakić I. Savić B. Švabić-Vlahović M. (2000). A modified microtiter-plate test for quantification of staphylococcal biofilm formation. J. Microbiol. Methods 40, 175–179. 10.1016/S0167-701200122-6 10699673

[B51] Tendero C. Tixier C. Tristant P. Desmaison J. Leprince P. (2006). Atmospheric pressure plasmas: a review. Spectrochim. Acta B 61, 2–30. 10.1016/j.sab.2005.10.003

[B52] Tomičić R. M. Cabarkapa I. S. Vukmirović Ã. M. Lević J. D. Tomičić Z. M. (2016). Influence of growth conditions on biofilm formation of *Listeria monocytogenes*. Food Feed. Res. 43, 19–24. 10.5937/FFR1601019T

[B53] Vleugels M. Shama G. Deng X. T. Greenacre E. Brocklehurst T. Kong M. G. (2005). Atmospheric plasma inactivation of biofilm-forming bacteria for food safety control. IEEE Trans. Plasma Sci. 33, 824–828. 10.1109/TPS.2005.844524

[B54] Yang L. Liu Y. Wu H. Høiby N. Molin S. Song Z. (2011). Current understanding of multi-species biofilms. Int. J. Oral Sci. 3, 74–81. 10.4248/IJOS11027 21485311 PMC3469880

[B55] Ziuzina D. Han L. Cullen P. J. Bourke P. (2015). Cold plasma inactivation of internalised bacteria and biofilms for *Salmonella enterica* serovar Typhimurium, *Listeria monocytogenes* and *Escherichia* coli. Int. J. Food. Microbiol. 210, 53–61. 10.1016/j.ijfoodmicro.2015.05.019 26093991

